# Development and validation of clinical criteria for critical illness-associated immune dysfunction: based on the MIMIC-IV database

**DOI:** 10.3389/fmed.2024.1465397

**Published:** 2024-12-11

**Authors:** Yanyou Zhou, Linfeng Tao, Shengsheng Yang, Juntu Li, Jun Liu

**Affiliations:** Department of Emergency and Critical Care Medicine, The Affiliated Suzhou Hospital of Nanjing Medical University, Suzhou Municipal Hospital, Gusu School of Nanjing Medical University, Suzhou Clinical Medical Center of Critical Care Medicine, Suzhou, China

**Keywords:** immune responses, critical immunological dysfunction, hyperinflammatory, immunosuppression, MIMIC-IV

## Abstract

**Background:**

Critical illness-associated immune dysfunction (CIID) is prevalent in the ICU and frequently resulted in uncontrollably immune responses. Critical immunological dysfunction is understood to be important, although there are currently no clinically accepted diagnostic criteria for it. Given this, we examined the literature and developed an initial diagnostic criterion that we validated using the MIMIC-IV database.

**Methods:**

We searched the related literature in the last 32 years. Patients admitted to the ICU for the first time were selected by screening the MIMIC-IV database. Different criteria were used to categorize patients into groups related to immune dysfunction (ID) and non-immune dysfunction (NID). Within the ID group, patients were subdivided into three subgroups: hyperinflammatory (HI), immunosuppression (IS), and a subgroup combining immunosuppression and hyperinflammation (HI+IS). The APACHE II was used to measure the patients’ severity. The association between immune dysfunction and mortality after 30 or 180 days was evaluated through the KM curves and COX regression analysis.

**Results:**

By summarizing relevant literature, we proposed the initial diagnostic criteria. The analysis included 43,965 patients, with approximately 77% meeting the diagnostic criteria for CIID. We observed that patients with immune dysfunction possessed higher APACHE II scores and there were differences in peak APACHE II among the three subgroups. When comparing patients’ 30-day mortality in the COX model, it is evident that patients in the IS subgroup had the lowest risk and patients in the HI subgroup the greatest risk after accounting for all covariates. In contrast, patients in the IS subgroup had the highest risk of death, those in the HI subgroup had the lowest risk when comparing long-term mortality. In summary, we propose and validate diagnostic criteria related to CIID. Subgroup analyses were carried out, which also revealed variations between the three groups.

**Conclusion:**

The diagnostic criteria were confirmed by the MIMIC-IV database, demonstrating the diagnostic criteria were scientifically valid and reliable.

## Introduction

Multiple Organ Dysfunction Syndrome (MODS) is a common and significant clinical syndrome in the ICU that can be caused by a range of reasons resulting in impaired function of the patient’s major organs such as heart, lung, and kidney. Unfortunately, when a patient proceeds to Multiple Organ Failure (MOF), the patient’s risk of death increases considerably (1, 2). In recent years, a new term has gradually come into everyone’s view – organ crosstalk. As the name suggests, organ crosstalk is the interaction and influence of different organs in physiological or pathological states, and the main organs involved are the heart, lungs, kidneys, liver and brain (3, 4). Taking the heart-kidney-lung axis as an example, there are structural similarities and close functional links between the heart, kidneys, and lungs, such as the activation of the classical pathway, the RAAS system, to maintain the stability of the internal environment (5). A multicenter cohort study showed that around 60% of patients with AKI in the ICU also had heart failure (6). Similarly, the risk of AKI in patients with heart failure is dramatically elevated (7). Another trial found that patients with ARDS were far more likely to develop AKI than patients without ARDS (8). Further, other investigations discovered that blocking the pathway of inflammatory chemokines can reduce the development of ARDS as a result of AKI (9). The substances that bind these organs together are chemical mediators and inflammatory cells present in the blood, such as NO, TNF-*α*, IL-6, and a range of inflammatory cytokines and immune cells that play a major role (10). These results suggest that the immune system plays an important role in the pathophysiology of dysfunction in all organ systems.

This immune response consists of pro-inflammatory and anti-inflammatory components (9). They are normally in harmony with one another; when they are not, the immune system gets dysregulated and immunological dysfunction happens (11). As the vigorous pro-inflammatory response is initiated, the anti-inflammatory response comes into play, potentially resulting in a down-regulation of inflammation and an inability to combat infections, ultimately leading to multi-organ dysfunction, multi-organ failure, and potentially death (12). Consequently, immune system disorder is a crucial component of multi-organ dysfunction and plays a significant role in disease progression. Despite increasing attention to the harm caused by immune system disorders, there are currently no established diagnostic criteria for critical illness-associated immune dysfunction (CIID). Therefore, based on a literature review, we have proposed preliminary diagnostic criteria for immune dysfunction associated with critical illness.

This study aims to evaluate the feasibility and utility of these proposed criteria, so that we can potentially improve recognition and intervention for critically ill patients. Additionally, assessing the correlation between these diagnostic criteria and disease severity as well as patient outcomes will provide valuable insights into the significance of immune dysfunction in the MODS.

## Method

### Literature search

To obtain preliminary diagnostic criteria, we searched for clinical English articles published between January 1, 1990, and December 31, 2022, on PubMed using Mesh search (keywords + free words): critical illness and (immune system diseases or immunosuppression therapy or immune system or immunity innate or adaptive immunity or inflammation or cytokine release syndrome or monitoring, immunologic or immunomodulation or immunotherapy). One hundred and twenty-six documents were selected by reading the titles and abstracts, followed by a reading of the full text (Supplementary Figure S1), resulting in 37 documents and preliminary diagnostic criteria.

### Data source

This diagnostic criterion was validated by clinical data from the Medical Information Mart for Intensive Care (MIMIC)-IV version 2.2, a publicly accessible ICU database that has detailed data on around 250,000 patients who were admitted in hospital and about 70,000 patients in the ICU between 2008 and 2019 (13). Researchers can filter and analyze various aspects of each patient’s medical records, such as demographic characteristics, vital signs, laboratory test results, and imaging examinations, using a unique code assigned to them upon admission. In order to gain access to this database, Author Zhou successfully completed online training courses and exams, obtaining certification number 53360516. As the data in this database is de-identified, informed consent from the patients was not required.

### Study population and criteria

**Figure 1:**
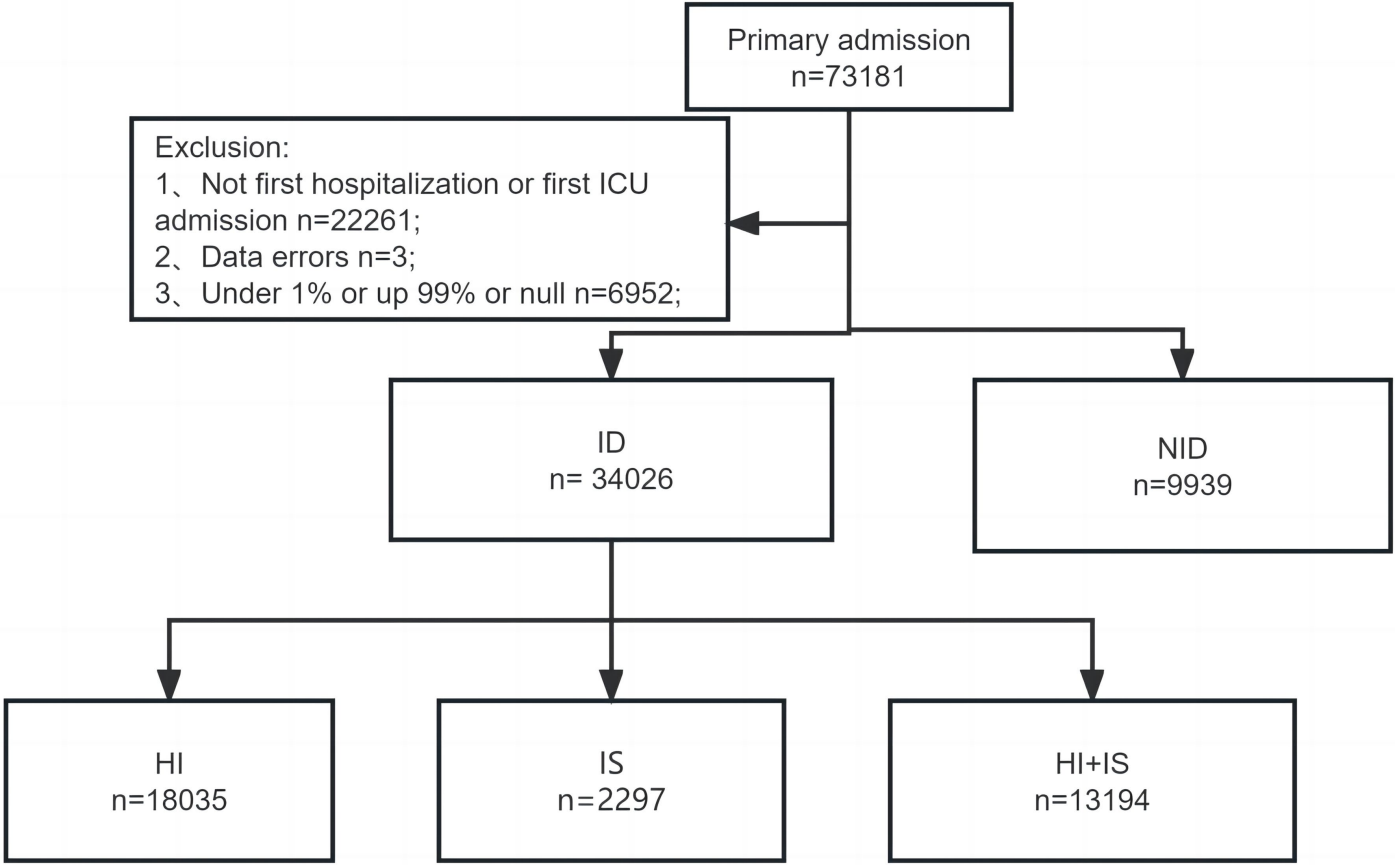
The flowchart for screening patients. ICU, intensive care unit; ID, immune dysfunction; NID, non-immune dysfunction; HI, hyperinflammation; SI, immunosuppression; HI + IS, combined hyperinflammation and immunosuppression.

A comprehensive analysis was conducted by examining the records of all adult individuals (18 years or older) who were admitted to ICU. For patients who had several hospitalizations, we only took into account data from the first hospital admission and first ICU stay to assure accuracy. Three individuals were eliminated from the analysis due to data errors, where the recorded time of death preceded the time of ICU admission. Patients with missing values were also excluded. Furthermore, to ensure the accuracy of subsequent analyses, patients with outlier values, defined as values exceeding the 99th percentile or falling below the 1st percentile, were removed. A detailed description of the screening process can be found in Figure 1.

### Covariates

In the current study, we collected data on various patient parameters, including (1) Demographic features (age, gender, race); (2) Acute Physiology and Chronic Health Evaluation II (APACHE II), Charlson Comorbidity Index; (3) Acute etiology (sepsis, septic shock, acute pancreatitis, acute myocardial infarction etc.); (4) Chronic comorbidities (HIV infection, diabetes, malignant cancer, congestive heart failure, hypertension, cerebrovascular disease); (5) Some vital signs and laboratory indicators (temperature, heart rate, respiratory rate, mean blood pressure, glucose, platelet, urea nitrogen); (6) The use of mechanical ventilation (MV) and renal replacement therapy (RRT) during ICU stay; (7) the length of ICU stay, 30-day mortality, 180-day mortality and ICU mortality of all patients. Furthermore, we collected vital signs and laboratory indicators from the initial dataset that was gathered upon the patient’s admission to the intensive care unit. We classified septic shock as sepsis accompanied by a mean blood pressure (MBP) of less than 65 mm Hg or the use of vasopressors such as norepinephrine, epinephrine, phenylephrine, and others (14). Besides, some variables, such as white blood cell (WBC), lactate and c-reactive protein (CRP) were deleted due to missing values >20%.

### Outcomes

Our primary end point was on determining the overall mortality rate within 30 days. Additionally, we assessed secondary outcomes – overall mortality rate within 180 days.

### Statistical analyses

We performed four normality tests to determine which normal distribution exists for continuous variables (Supplementary Table S1). The data for continuous variables were represented as median (interquartile ranges, IQR) due to non-normal distribution, while those for categorical data were represented as frequencies. The Kruskal–Walli’s test was used for continuous variables, and the *χ*^2^ test or Fisher’s exact test was used for categorical variables. The APACHE II score was employed as an assessment of the severity of the disease, and the findings were depicted in the form of line graphs.

The Kaplan–Meier (KM) method draws a cumulative incidence curve, indicating the occurrence of ICU and in hospital deaths in different groups of patients, and the differences in risk between the groups were compared using log-rank tests. The potential factors that could affect the results were taken into account and adjusted for in four different models. In the initial phase of our analysis, we conducted intergroup comparisons without taking into account any additional variables, which we labeled as Model 1. Then we made age, gender, and race adjustments (Model 2). Additionally, we made adjustments for several illness severity ratings (APACHE II and Charlson Comorbidity Index) as well as associated etiology and comorbidities, like diabetes and septic shock (Models 3). The remaining factors are then modified (Models 4).

Furthermore, one-factor COX regression was performed to determine the connection between immune dysfunction and ICU mortality. We also evaluated the multicollinearity between the variables using variance inflation factors (VIF). Supplementary Table S2 revealed that the VIF of each variable was less than 4, indicating that there was no multicollinearity between them. In addition, we performed calculations for the Concordance Index (C-index) to assess the performance of the model and make comparisons between multiple models.

The *p*-values with less than 0.05 were taken as the statistically significant (two-sides). Navicat Premium 16.0 software was used for data extraction, and analysis of statistical data was conducted with the R software (version 4.3) or Empowerstats (version 4.1).

## Results

### Construction of diagnostic criteria

The search yielded 21,658 relevant publications initially, followed by 6,657 publications after excluding non-English articles, non-human clinical trials or reviews, and publications not from the period 1990 to 2022. One hundred and twenty-six documents were selected by reading the titles and abstracts, followed by a reading of the full text, resulting in 36 documents and preliminary diagnostic criteria (Table 1).

**Table 1:** Diagnostic criteria for critical illness-related immune dysfunction (CIID).

	CIID
1. Trigger factors	A known infection and/or non-infectious clinical insult^a^
2. Hyperinflammation	SIRS≥2
3. Organ dysfunction	An acute increase in total SOFA score 2 points consequent to the infection and/or non-infectious insults
4. Immunosuppression	Any one of the following: ① absolute neutrophil value <1.5 × 10^9^/L; ② absolute lymphocyte value <1.2 × 10^9^/L; ③ HLA-DR < 80% or <20,000 Ab/cell; ④ LPS-TNF-*α* < 250 pg./mL.
5. Secondary infection	Any new-onset infection (with likelihood possible, probable, or definite) starting more than 48 h after ICU admission for which the clinical team started a new antibiotic regimen.

Diagnostic criteria of CIID: 1 plus any one or more of the other criteria; HI: Both Satisfied both points 2 and 3; SI: Satisfied point 4 or 5. SIRS, Systemic inflammatory response syndrome; SOFA, Sequential Organ Failure Assessment; HLA-DR, monocytic human leukocyte antigen-DR; LPS-TNF, Lipopolysaccharide tumor necrosis factor.^a^Common risk factors for CIID include sepsis, major trauma, severe burns, shock, pancreatitis, malnutrition, acquired immune diseases, diabetes, cancer, iatrogenic factors, heat stroke, drowning and so on.

First, all patients admitted to the ICU were determined as the critical illness. According to the criteria, we also separated the patients into two groups: immune dysfunction (ID) group and non-immune dysfunction (NID) group. Additionally, within the ID group, we identified three subgroups. Patients who met the second and third criteria were classified into the hyperinflammation (HI) subgroup, while those who met the fourth or fifth criteria were placed in the immunosuppression (IS) subgroup. Finally, patients who met all the aforementioned criteria were categorized into the combined hyperinflammation and immunosuppression (HI + IS) subgroup.

### Baseline characteristics

After a series of screenings, we were left with a final cohort of 43,965 patients for further analysis. A total of 9,939 (22.61%) of them were categorized as belonging to the NID group. In contrast, there were 34,026 patients (77.39%) who met the criteria for being the ID group and were included in our analysis accordingly. Meanwhile, the HI group has 18,035 (53.00%) patients, while the IS group has 2,797 (8.22%) and the HI + IS group has 13,194 (38.78%) individuals, respectively.

The detailed information for each variable can be found in Table 2. Most of the covariates have significant differences between them apart from race.

**Table 2:** Baseline demographic and clinical characteristics.

Feature	NID *N* = 9,939	ID *N* = 34,026	*P*-value	HI *N* = 18,035	IS *N* = 2,797	HI + IS *N* = 13,194	*P*-value
Demographic feature							
Age	62.00 (49.00–74.00)	66.00 (54.00–77.00)	<0.001	66.00 (54.00–77.00)	66.00 (54.00–76.00)	67.00 (55.00–78.00)	<0.001
Gender			<0.001				<0.001
Male	5,175 (52.07%)	19,577 (57.54%)		10,699 (59.32%)	1,532 (54.77%)	7,346 (55.68%)	
Female	4,764 (47.93%)	14,449 (42.46%)		7,336 (40.68%)	1,265 (45.23%)	5,848 (44.32%)	
Race			0.149				0.063
White	3,184 (32.04%)	11,163 (32.81%)		5,819 (32.27%)	928 (33.18%)	4,416 (33.47%)	
Other	6,755 (67.96%)	22,863 (67.19%)		12,216 (67.73%)	1869 (66.82%)	8,778 (66.53%)	
Scoring							
APACHE II	12.00 (9.00–17.00)	19.00 (15.00–24.00)	<0.001	18.00 (14.00–23.00)	15.00 (11.00–20.00)	21.00 (16.00–26.00)	<0.001
Charlson	4.00 (3.00–6.00)	5.00 (4.00–7.00)	<0.001	5.00 (3.00–7.00)	5.00 (4.00–8.00)	6.00 (4.00–8.00)	<0.001
Etiology							
Sepsis	907 (9.13%)	18,569 (54.57%)	<0.001	8,297 (46.00%)	762 (27.24%)	9,510 (72.08%)	<0.001
Septic shock	246 (2.48%)	8,319 (24.45%)	<0.001	3,359 (18.62%)	283 (10.12%)	4,677 (35.45%)	<0.001
Acute pancreatitis	89 (0.90%)	618 (1.82%)	<0.001	177 (0.98%)	49 (1.75%)	392 (2.97%)	<0.001
Myocardial infarct	907 (9.13%)	4,466 (13.13%)	<0.001	3,116 (17.28%)	438 (15.66%)	2,170 (16.45%)	<0.001
Comorbidity							
HIV_infection	56 (0.56%)	278 (0.82%)	<0.001	107 (0.59%)	40 (1.43%)	131 (0.99%)	<0.001
ARDS	469 (4.72%)	6,681 (19.63%)	<0.001	1939 (10.75%)	500 (17.88%)	4,242 (32.15%)	<0.001
AKF	600 (6.04%)	5,932 (17.43%)	<0.001	2,249 (12.47%)	371 (13.26%)	3,312 (25.10%)	<0.001
Diabetes	1,428 (14.37%)	5,982 (17.58%)	<0.001	3,199 (17.74%)	452 (16.16%)	2,331 (17.67%)	<0.001
Hypertension	4,509 (45.37%)	15,019 (44.14%)	0.03	8,363 (46.37%)	1,207 (43.15%)	5,449 (41.30%)	<0.001
Cerebrovascular disease	681 (6.85%)	776 (2.28%)	<0.001	471 (2.61%)	58 (2.07%)	247 (1.87%)	<0.001
Congestive failure	1,567 (15.77%)	8,868 (26.06%)	<0.001	4,275 (23.70%)	717 (25.63%)	3,876 (29.38%)	<0.001
Malignant cancer	907 (9.13%)	4,466 (13.13%)	<0.001	1942 (10.77%)	432 (15.45%)	2092 (15.86%)	<0.001
Vital signs and laboratory indicators							
Temperature	37.10 (36.90–37.40)	37.40 (37.10–38.10)	<0.001	37.30 (37.10–37.80)	37.30 (37.00–37.80)	37.70 (37.20–38.50)	<0.001
HR	93.00 (84.00–107.00)	109.00 (96.00–125.00)	<0.001	106.00 (95.00–119.00)	99.00 (87.00–116.00)	117.00 (103.00–132.00)	<0.001
RR	26.00 (23.00–30.00)	30.00 (26.00–35.00)	<0.001	29.00 (25.00–33.00)	28.00 (24.00–33.00)	32.00 (28.00–38.00)	<0.001
MBP	103.00 (93.00–116.00)	104.00 (91.00–118.00)	0.019	100.00 (88.00–115.00)	105.00 (93.00–120.00)	108.00 (95.00–123.00)	<0.001
PLT	176.00 (138.00–219.00)	134.00 (94.00–183.00)	<0.001	138.00 (103.00–185.00)	156.00 (110.00–201.00)	122.00 (76.00–175.00)	<0.001
BUN	20.00 (15.00–30.00)	29.00 (20.00–50.00)	<0.001	26.00 (18.00–41.00)	27.00 (19.00–45.00)	36.00 (23.00–63.00)	<0.001
Glucose	143.00 (114.00–188.00)	177.00 (142.00–228.00)	<0.001	172.00 (139.00–216.00)	161.00 (127.00–218.00)	188.00 (149.00–251.00)	<0.001
Curing							
Ventilation	5,197 (52.29%)	28,248 (83.02%)	<0.001	14,561 (80.74%)	2014 (72.01%)	11,673 (88.47%)	<0.001
RRT	127 (1.28%)	1921 (5.65%)	<0.001	597 (3.31%)	118 (4.22%)	1,206 (9.14%)	<0.001
Outcome							
Mortality of 30 days	283 (2.85%)	4,471 (13.14%)	<0.001	1771 (9.82%)	237 (8.47%)	2,463 (18.67%)	<0.001
Mortality of 180 days	746 (7.51%)	7,394 (21.73%)	<0.001	2,853 (15.82%)	549 (19.63%)	3,992 (30.26%)	<0.001
Mortality of ICU	6 (0.06%)	89 (0.26%)	<0.001	23 (0.13%)	2 (0.07%)	64 (0.49%)	<0.001

Kruskal–Walli’s test was used for continuous variables, and the *χ*^2^ test or Fisher’s exact test was used for categorical variables.

Median (interquartile ranges, IQR) for continuous variables: the *P*-value was calculated by the Kruskal–Wallis test; Frequency (percentage) for categorical variables: the *P*-value was calculated by the *χ*^2^ test or Fisher’s exact test; ID, immune dysfunction; NID, non-immune dysfunction; HI, hyperinflammation; SI, immunosuppression; HI + IS, combined hyperinflammation and immunosuppression; APACHE II, Acute Physiology and Chronic Health Evaluation II; ARDS, acute respiratory distress syndrome; AKF, acute kidney injury; BUN, blood urea nitrogen; HR, heart rate; MBP, mean blood pressure; PLT, platelet; RR, respiratory rate; RRT, renal replacement therapy.

It is worth noting that the median age of patients in the ID group was slightly higher compared to the NID group, although the difference between the three subgroups was small. In terms of etiology, it was discovered that infections like sepsis accounted for a bigger percentage of patients in the ID group (54.57%), while that percentage reached as high as 77% in the HI + IS group. Additionally, with the exception of patients with hypertension, the proportion of patients in the ID group was always larger than the proportion of patients in the NID group, regardless of comorbidity or whether they were admitted to the ICU with mechanical ventilation or RRT.

### Disease severity grading

Supplementary Figure S2A clearly shows the gap in number distribution between the two groups. The NID group consists primarily of patients with scores less than 20, whereas the ID had higher scores. Moving on, subgroup comparisons in Supplementary Figure S2B show that the majority of patients in all three subgroups have higher APACHE II scores than the NID. Notably, patients in the IS subgroup had significantly lower peak scores, whereas patients in the HI + IS subgroup have the highest peak scores.

### Survival analysis

**Figure 2:**
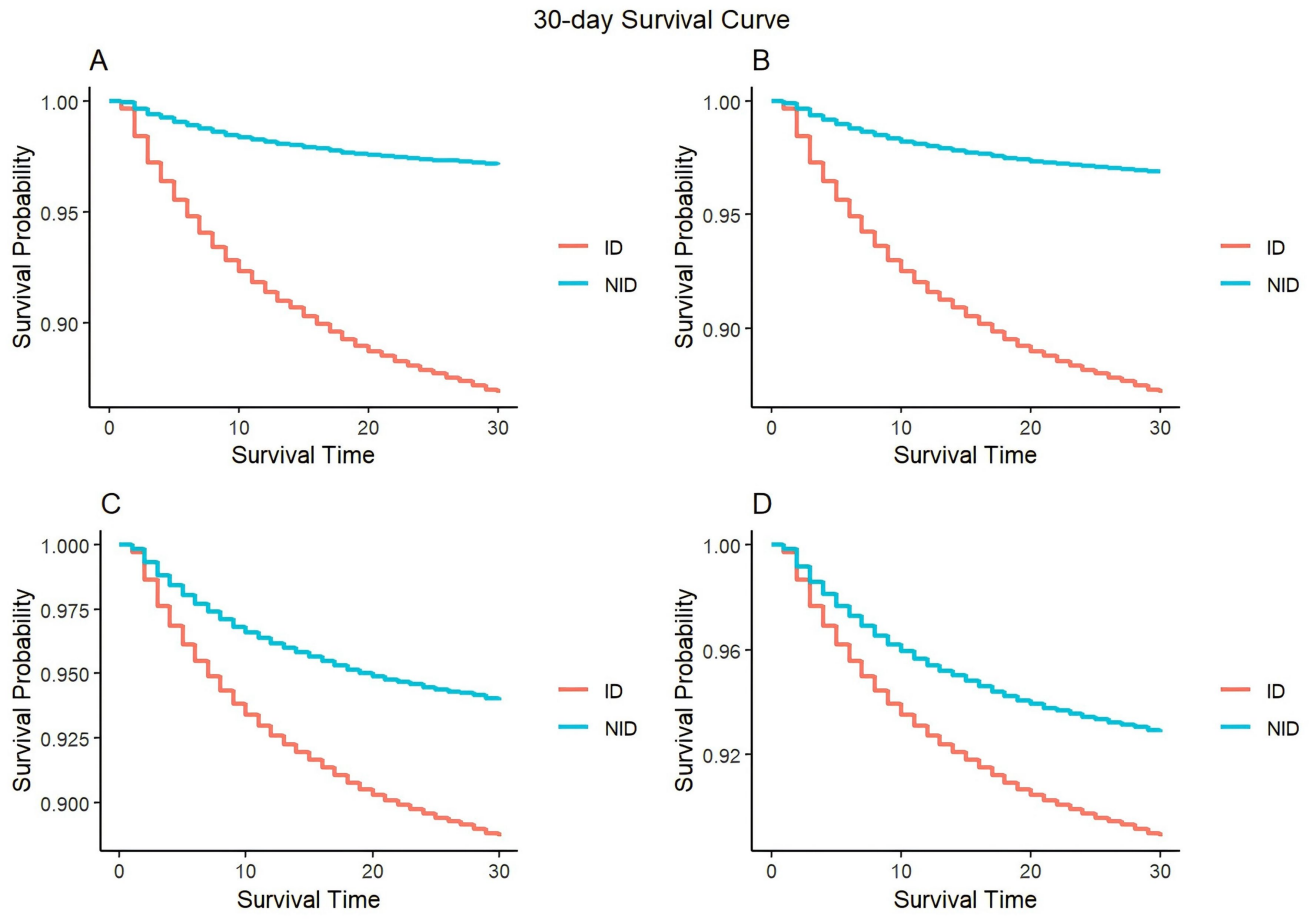
Kaplan–Meier survival curves of 30-day mortality between the ID and NID group. **(A)** Model 1 (uncorrected variable); **(B)** model 2 (adjusted for age, gender, and race); **(C)** model 3 (adjusted for age, gender, race, APACHE II, Charlson, sepsis, septic shock, acute pancreatitis, myocardial infarct, HIV infection, ARDS, AKF, diabetes, hypertension, cerebrovascular disease, congestive failure, and malignant cancer); **(D)** model 4 (adjusted for all variable).

During our analysis of the KM curve, we noticed significant discrepancies in the survival rates between the ID and the NID group at both the 30-day and 180-day marks (Figure 2 and Supplementary Figure S3). In both short-term and long-term mortality, we can see that patients in the ID always have a higher mortality rate than those in the NID. And with the gradual correction of covariates, it can be seen that the survival rate increases in both groups, but the survival rate of patients in the ID shows a substantial increase. Of these, the most significant reduction in patient mortality was seen when correcting for scores and associated comorbidities.

Similar results can be found in the survival curves of subgroups (Supplementary Figures S4, S5). When uncorrected for all confounders, the HI + IS group’s curve for short-term survival decreases the most and has a greater mortality rate (Supplementary Figure S4A). The HI + IS group’s curve was gradually brought closer to the HI curve as factors were corrected over time (Supplementary Figures S4B,C), and when all covariates were taken into account (Supplementary Figure S4D), patients in the HI were shown to have a greater death rate. In terms of long-term mortality (Supplementary Figure S5), adjusting for covariates causes all four curves to increase and come closer together. Interestingly, patients in the HI had a greater survival rate than those in the other two subgroups, while those in the IS had a higher mortality rate (Supplementary Figure S5D).

### COX proportional-hazards regression model

When COX analyses were performed (Table 3), after correcting for all covariates, patients within the ID have a significantly higher susceptibility to death and short-term mortality compared to patients within the NID. When subgroups were compared, it was discovered that patients in the HI (1.72 95%CI: 1.50–1.96) had the highest risk of dying, followed by those in the HI + IS (1.65 95%CI: 1.43–1.89), and those in the IS (1.58 95%CI: 1.32–1.88) had the lowest risk. The outcomes were reversed when it came to the risk of long-term mortality. Patients in the HI (1.31 95%CI: 1.20–1.43) had the lowest risk of mortality among the three groups, while those in the IS (1.62 95%CI: 1.45–1.81) had the highest risk.

**Table 3:** The COX analysis in different groups and subgroups.

ID				
	30 days		180 days	
	HR (95%CI)	*P*	HR (95%CI)	*P*
M1	4.87 (4.32, 5.49)	<0.0001	3.17 (2.94, 3.42)	<0.0001
M2	4.40 (3.91, 4.97)	<0.0001	2.88 (2.67, 3.10)	<0.0001
M3	2.05 (1.81, 2.33)	<0.0001	1.57 (1.45, 1.70)	<0.0001
M4	1.68 (1.48, 1.91)	<0.0001	1.40 (1.29, 1.52)	<0.0001

	HI			
	30 days		180 days	
	HR (95%CI)	*P*	HR (95%CI)	*P*
M1	3.61 (3.19, 4.10)	<0.0001	2.25 (2.07, 2.43)	<0.0001
M2	3.31 (2.92,3.75)	<0.0001	2.05 (1.89, 2.22)	<0.0001
M3	2.09 (1.84, 2.38)	<0.0001	1.45 (1.33, 1.57)	<0.0001
M4	1.72 (1.50, 1.96)	<0.0001	1.31 (1.20, 1.43)	<0.0001

	IS			
	30 days		180 days	
	HR (95%CI)	*P*	HR (95%CI)	*P*
M1	3.03 (2.55, 3.60)	<0.0001	2.76 (2.47, 3.09)	<0.0001
M2	2.77 (2.33,3.30)	<0.0001	2.54 (2.28, 2.84)	<0.0001
M3	1.66 (1.39, 1.97)	<0.0001	1.65 (1.47, 1.84)	<0.0001
M4	1.58 (1.32, 1.88)	<0.0001	1.62 (1.45, 1.81)	<0.0001

	HI + IS			
	30 days		180 days	
	HR (95%CI)	*P*	HR (95%CI)	*P*
M1	7.04 (6.22, 7.96)	<0.0001	4.63 (4.29, 5.01)	<0.0001
M2	6.26 (5.54,7.08)	<0.0001	4.15 (3.84, 4.49)	<0.0001
M3	2.13 (1.87, 2.44)	<0.0001	1.78 (1.64, 1.95)	<0.0001
M4	1.65 (1.43, 1.89)	<0.0001	1.53 (1.39, 1.67)	<0.0001

ID, immune dysfunction; NID, non-immune dysfunction; HI, hyperinflammation; SI, immunosuppression; HI + IS, combined hyperinflammation and immunosuppression.

M1: uncorrected variable.

M2: adjusted for age, gender, race.

M3: adjusted for age, gender, race, APACHE II, Charlson, sepsis, septic shock, acute pancreatitis, myocardial infarct, HIV infection, ARDS, AKF, diabetes, hypertension, cerebrovascular disease, congestive failure, malignant cancer.

M4: adjusted for all variable.

In the subsequent C-index calculations of the models for each group, we observed a gradual increase in the C-index values as we continued to correct the covariates (Supplementary Table S3). When all the covariates were corrected, we noticed that the C-index for short-term immune dysfunction with non-immune dysfunction was the highest (0.822 se: 0.003), followed by long-term immune dysfunction (0.821 se: 0.003). Additionally, during the subgroup comparison, we found that the C-index of the model was 0.796 (se: 0.002) for short-term immune dysfunction and 0.797 (se: 0.002) for long-term immune dysfunction. These results demonstrate that the model exhibits a strong predictive performance.

After controlling for all confounders, we discovered that the C-index for short-term immunological dysfunction with non-immune dysfunction was the greatest, followed by long-term immune dysfunction. Furthermore, we discovered that the model’s C-index was 0.796 for short-term immunological dysfunction and 0.797 for long-term immune dysfunction during the subgroup comparison. These findings show that the model performs well in terms of prediction.

## Discussion

After searching and reading a large amount of literature, we proposed preliminary diagnostic criteria for CIID and validated them using the MIMIC IV database. According to the data, a large majority of ICU patients experienced immune dysfunction, which was primarily caused by infection. As the patients’ condition deteriorated, their chances of death climbed proportionally. When several variables were considered, it was discovered that patients in the HI had a higher risk of short-term mortality. Furthermore, patients in the IS group had a higher short-term mortality risk, whereas patients in the IS group had a higher long-term mortality risk.

Previous research has revealed that the mortality rate of MODS in septic patients can be as high as 40% (15). It can also be seen in this study that patients with CIID accounted for most. Therefore, we believe in the prevalence of immune dysfunction in critically ill patients and the need for early recognition and intervention by healthcare professionals.

The APACHE II score is a widely recognized and frequently employed measure in the ICU to assess the severity of a patient’s illness. When critically ill patients have higher APACHE II scores as well as comorbidities, the sicker the patient is and the increased risk of death (16). Higher APACHE II scores and underlying health issues in critically sick individuals suggest a higher level of illness and an increased chance of mortality. An international investigation including numerous hospitals discovered a link between the severity of a patient’s condition and the chance of infection. In other words, the more a patient’s severe illness, the greater the chance of infection. This study also discovered that organisms in critically ill individuals with infections are more likely to induce additional health concerns, such as comorbidities (17). Furthermore, a multi-center retrospective investigation verified the presence of neutropenia in patients with cancer, with the development of neutropenic small bowel colitis and an increased risk of fungal invasion (18).

When an organism causes an infection, the death rate of patients might nearly triple if timely care is not implemented (19). For this reason, we believe that the activation of the body’s immune system happens in response to both infections and non-infections and consequent antagonism between pro-inflammatory and anti-inflammatory responses, leading to impaired immune function, a lack of circulating T, B lymphocytes (20, 21), and the emergence of a phase of persistent inflammation, immunosuppression and catabolism (PICS) (22). In addition, we have found that patients have longer hospital stays and have a worse clinical prognosis when they have PICS (23). Otto et al. (24) has been verified that, people in the later stages of severe sickness have immunosuppression, which makes them more susceptible to infections and increases their chance of death.

The now generally accepted view is that it is the result of the interaction between the body’s systemic inflammatory response syndrome (SIRS) and the compensatory anti-inflammatory response syndrome (CARS) (25). Activation of the body’s innate immune system during the early stages of the disease leads to the activation of a considerable number of intrinsic immune cells, which produce a slew of pro-inflammatory mediators. While the body does generate anti-inflammatory mediators during this time, SIRS is primarily responsible for the pro-inflammatory response, or even hyperinflammation (26). Immune cell apoptosis and pro-inflammatory factor depletion describe the stage of immunosuppression in the body, which is typically characterized by a decrease in the absolute values of neutrophils and lymphocytes and a decrease in the expression of HLA-DR secreted by monocytes (26–28).

Our study found similar results, indicating that hyperinflammation dominates short-term survival, with a larger probability of death. Long-term survival, on the other hand, is controlled by immunosuppression. Patients who have both hyperinflammation and immunosuppression have risk factors that are similar to the other two groups, but are substantially higher when other factors are not taken into account. We suspect that these people have other underlying disorders that contribute to the condition’s aggravation.

Measuring the indicators related to immune dysfunction in critically ill patients in this study is simple and easy to obtain in daily clinical work, and it is easy to be understood and used by clinicians, which makes it practical and applicable. Secondly, the COX model was constructed by grouping critically ill patients in this study and the C-index test was conducted, which revealed that the model has a certain degree of reliability. The subgroups also showed that critically ill patients with different subtypes of immune dysfunction had different prognoses; for example, patients in the hyperinflammatory group were more likely to die early in life, whereas patients in the immunosuppressed group showed a higher risk of death later in life. Different subtypes of immune dysfunction have different treatment strategies. When the patient is in SIRS, the main strategy is anti-inflammatory, whereas when the patient is in the immunosuppressed phase, it is to enhance immunity and reduce the likelihood of secondary infections in the organism.

The primary implication of our findings is to define patients with the CIID and to provide different immunotherapy directions according to different subtypes. However, certain limits must be acknowledged. To begin, the lack of a globally acknowledged definition required the use of a somewhat arbitrary term in this study. Second, while HLA-DR and LPS-TNF-*α* can be useful indicators for diagnosing immunosuppression (28, 29), measuring it proved difficult due to the lack of routine tests and the lack of reliable values in the database. As a result, HLA-DR and LPS-TNF-α were not used as immunosuppressive indication in this study’s analysis, and we maybe ignore some patients who met the immunosuppression criteria. Third, this investigation was carried out retrospectively in a single-center context, using data gathered during patients’ hospitalizations. As a result, considerable gaps in the data for key critical variables existed, resulting to increased inaccuracy in the experimental results. As a result, we predict that future advances in medical research will permit the use of HLA-DR as a widely used clinical indicator for early patient stage determination and the deployment of targeted immunotherapy treatments.

## Conclusion

Critical illness-associated immune dysfunction is common in critically ill patients, and this analysis also found that the majority of the patients in this study had immunological dysfunction. These findings strongly show that immunological dysfunction, on its own, can have a considerable impact on patient survival. As a result, it is critical to regularly monitor and assess patients’ dynamic changes in immune function. This will allow for the prompt delivery of targeted medicines to address any changes in the patient’s immune system.

## Figures and Tables

**Table:** 

APACHE II	Acute Physiology and Chronic Health Evaluation II
ARDS	Acute respiratory distress syndrome
AKI	Acute kidney injury
BUN	Blood urea nitrogen
CARS	Compensatory anti-inflammatory response syndrome
C-index	Concordance Index
CIID	Critical illness-associated immune dysfunction
CRP	C-reactive protein
HI	Hyperinflammation
HI + IS	Hyperinflammation and immunosuppression
HLA-DR	Human leukocyte antigen-DR
HR	Heart rate
ICU	Intensive care unit
ID	Immune dysfunction
KM	Kaplan–Meier
MBP	Mean blood pressure
MIMIC	Medical Information Mart for Intensive Care
MODS	Multiple Organ Dysfunction Syndrome
MOF	Multiple Organ Failure
MV	Mechanical ventilation
NID	Non-immune dysfunction
PLT	Platelet
SI	Immunosuppression
SIRS	Systemic inflammatory response syndrome
SOFA	Sequential Organ Failure Assessment
RR	Respiratory rate
RRT	Renal replacement therapy
VIF	Variance inflation factors
WBC	White blood cell

## Data Availability

The original contributions presented in the study are included in the article/Supplementary material, further inquiries can be directed to the corresponding author.
