# Supplementary material for: Development and validation of clinical criteria for critical illness-associated immune dysfunction: based on the MIMIC-IV database
Source: Front Med (Lausanne). 2024 Dec 11;11:1465397. doi: 10.3389/fmed.2024.1465397 (PMC11707548; doi:10.3389/fmed.2024.1465397)
Supplement: Supplementary file 1 [file Data_Sheet_1.docx]

Supplementary Table S1: The outcomes of four normality tests to determine which normal distribution exists for continuous variables.

| 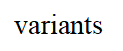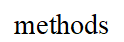 | Anderson-Darling normality test | Cramer-von Mises normality test | Lilliefors (Kolmogorov-Smirnov) normality test | Pearson chi-square normality test |
| --- | --- | --- | --- | --- |
| AGE | P <0.000001 | P <0.000001 | P <0.000001 | P <0.000001 |
| APACHEII | P <0.000001 | P <0.000001 | P <0.000001 | P <0.000001 |
| CHARLSON | P <0.000001 | P <0.000001 | P <0.000001 | P <0.000001 |
| PLT | P <0.000001 | P <0.000001 | P <0.000001 | P <0.000001 |
| BUN | P <0.000001 | P <0.000001 | P <0.000001 | P <0.000001 |
| HR | P <0.000001 | P <0.000001 | P <0.000001 | P <0.000001 |
| MBP | P <0.000001 | P <0.000001 | P <0.000001 | P <0.000001 |
| RR | P <0.000001 | P <0.000001 | P <0.000001 | P <0.000001 |
| GLUCOSE | P <0.000001 | P <0.000001 | P <0.000001 | P <0.000001 |
| TEMPERATURE | P <0.000001 | P <0.000001 | P <0.000001 | P <0.000001 |

APACHE II, Acute Physiology and Chronic Health Evaluation II; PLT, platelet; BUN, blood urea nitrogen; HR, heart rate; MBP, mean blood pressure; RR, respiratory rate; RRT, renal replacement therapy.

Supplementary Table S2: The VIF of each variable that was less than 4.

| FACTORS | VIF |
| --- | --- |
| GENDER | 1.1 |
| AGE | 2.2 |
| RACE | 1 |
| APACHEII | 2.1 |
| CHARLSON | 3 |
| SEPSIS | 1.7 |
| HIV INFECTION | 1.1 |
| ARF | 1.2 |
| AKF | 1.2 |
| DIABETES | 1.3 |
| HYPERTENION | 1.2 |
| ACUTE PANCREATITIS | 1 |
| CEREBROVASCULAR DISEASE | 1 |
| CONGESTIVE HEART FAILURE | 1.4 |
| MALIGNANT CANCER | 1.4 |
| MYOCARDIAL INFARCT | 1.2 |
| SPETIC SHOCK | 1.6 |
| PLT | 1.2 |
| BUN | 1.7 |
| HR | 1.4 |
| MBP | 1.2 |
| RR | 1.4 |
| GLUCOSE | 1.3 |
| TEMPERATURE | 1.4 |
| VENTILATION | 1.2 |
| RRT | 1.2 |

APACHE II, Acute Physiology and Chronic Health Evaluation II; ARDS, acute respiratory distress syndrome; AKF, acute kidney injury; BUN, blood urea nitrogen; HR, heart rate; MBP, mean blood pressure; PLT, platelet; RR, respiratory rate; RRT, renal replacement therapy.

Supplementary Table S3: The C-index values.

|  | NID VS ID | | subgroup | |
| --- | --- | --- | --- | --- |
|  | 30 days | 180days | 30 days | 180 days |
| M1 | 0.589(0.002) | 0.576(0.002) | 0.651(0.003) | 0.636(0.003) |
| M2 | 0.692(0.004) | 0.683(0.003) | 0.715(0.003) | 0.706(0.003) |
| M3 | 0.809(0.003) | 0.791(0.002) | 0.809(0.003) | 0.792(0.002) |
| M4 | 0.822(0.003) | 0.796(0.002) | 0.821(0.003) | 0.797(0.002) |

ID, immune dysfunction; NID, non-immune dysfunction; M1:uncorrected variable;

M2:adjusted for age, gender, race;

M3:adjusted for age, gender, race, APACHE II, charlson, sepsis, spetic shock, acute pancreatitis, myocardial infarct, HIV infection, ARDS, AKF, diabets, hypertenion, cerebrovascular disease, congestive failure, malignant cancer;

M4 :adjusted for all variable.


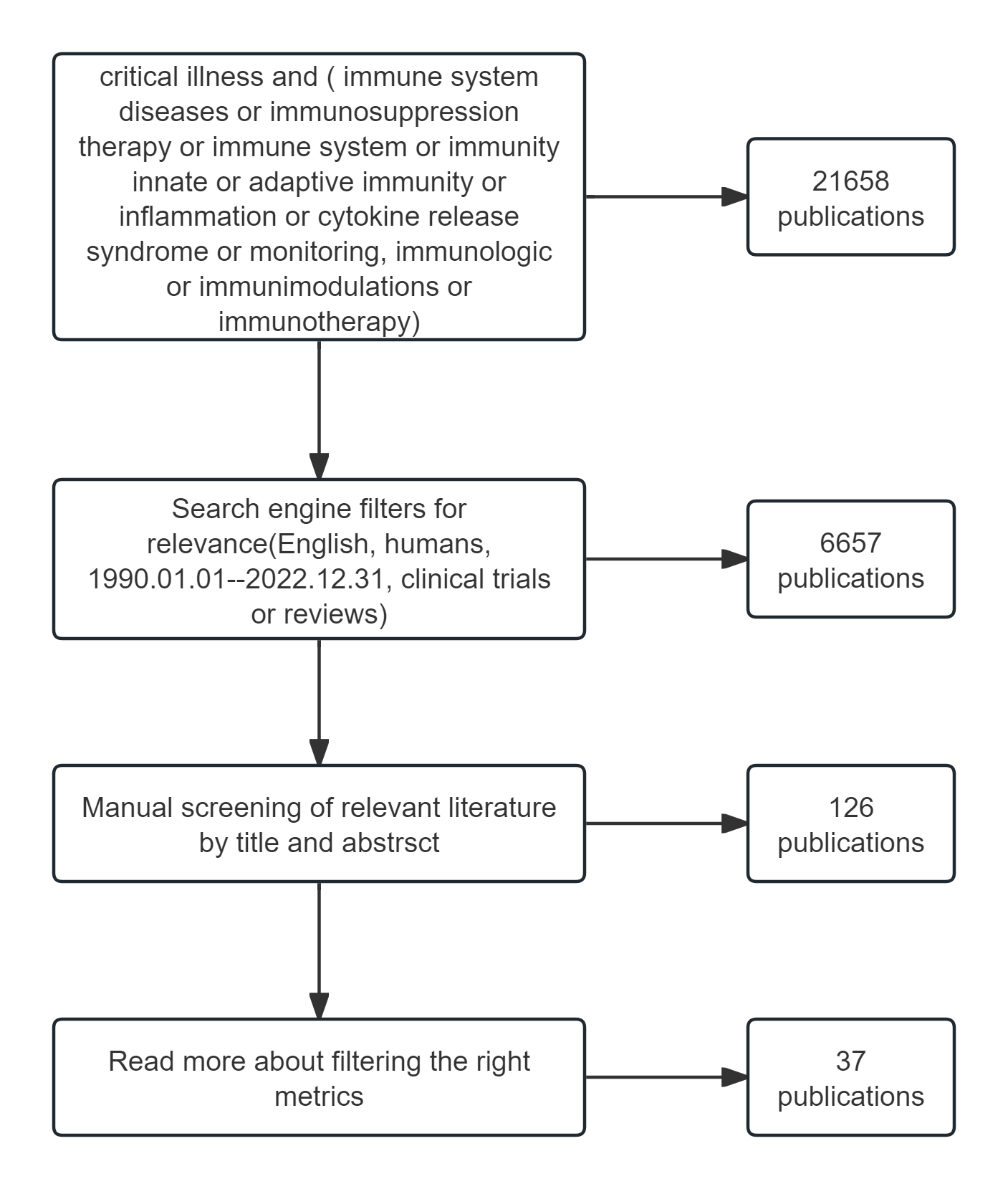


Supplementary Figure1: Literature search flowchart.


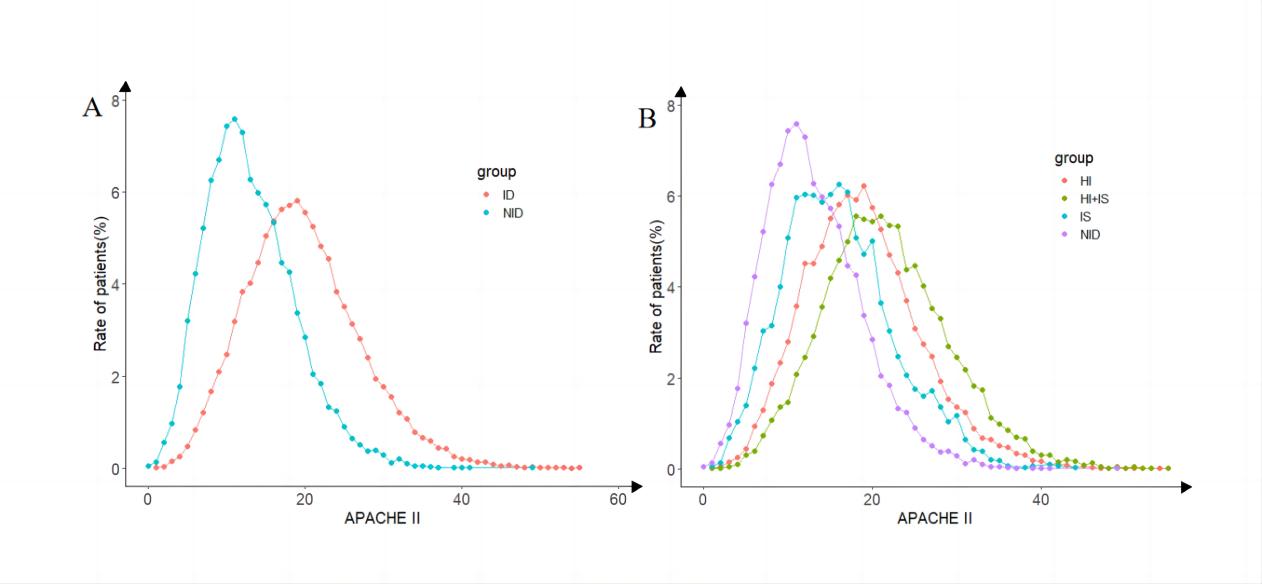


Supplementary Figure 2: The relationship between APACHE II and patient distribution between groups. ID, immune dysfunction; NID, non-immune dysfunction; HI, hyperinflammation; SI, immunosuppression; HI+IS, combined hyperinflammation and immunosuppression.


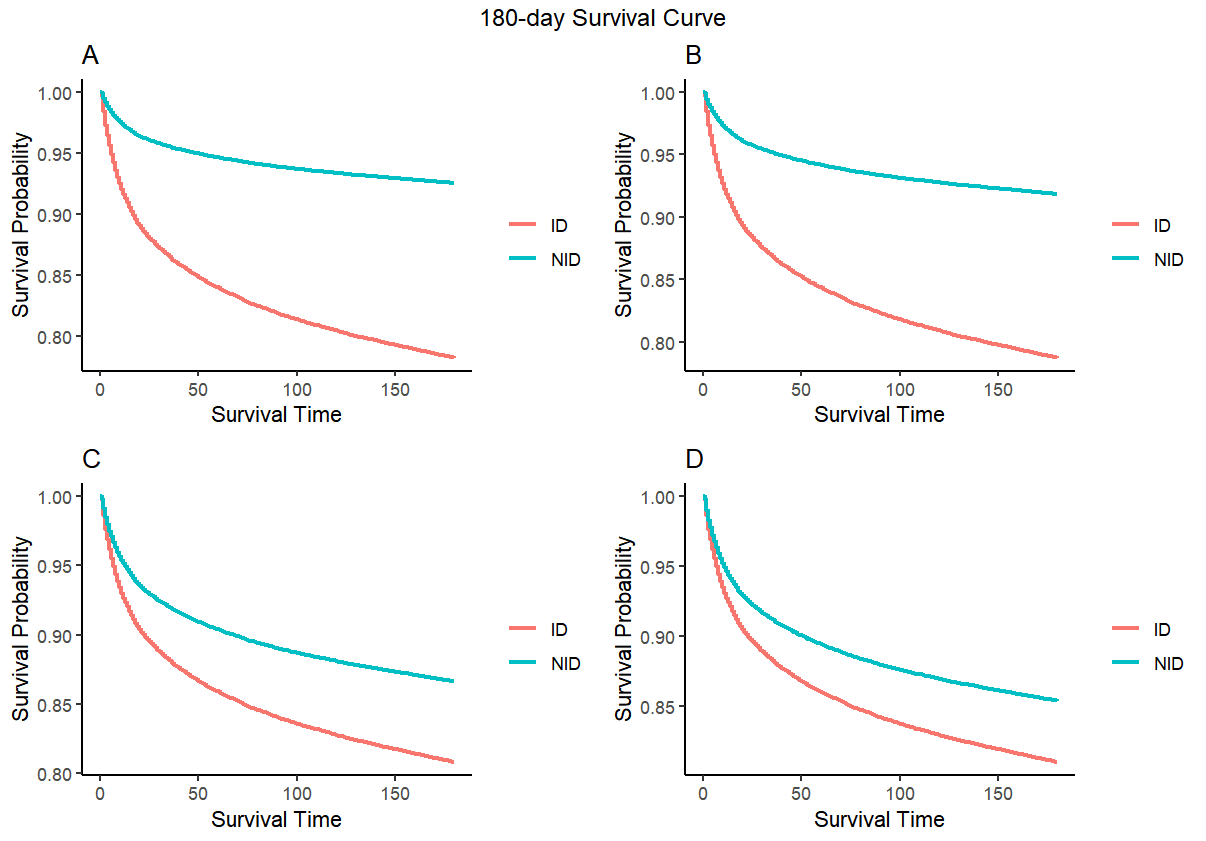


Supplementary Figure 3: Kaplan-Meier survival curves of 180-day mortality between the ID and NID group.A: model 1 (uncorrected variable); B: model 2 (adjusted for age, gender, race); C: model 3 (adjusted for age, gender, race, APACHE II, charlson, sepsis, spetic shock, acute pancreatitis, myocardial infarct, HIV infection, ARDS, AKF, diabets, hypertenion, cerebrovascular disease, congestive failure, malignant cancer; D: model 4 (adjusted for all variable).


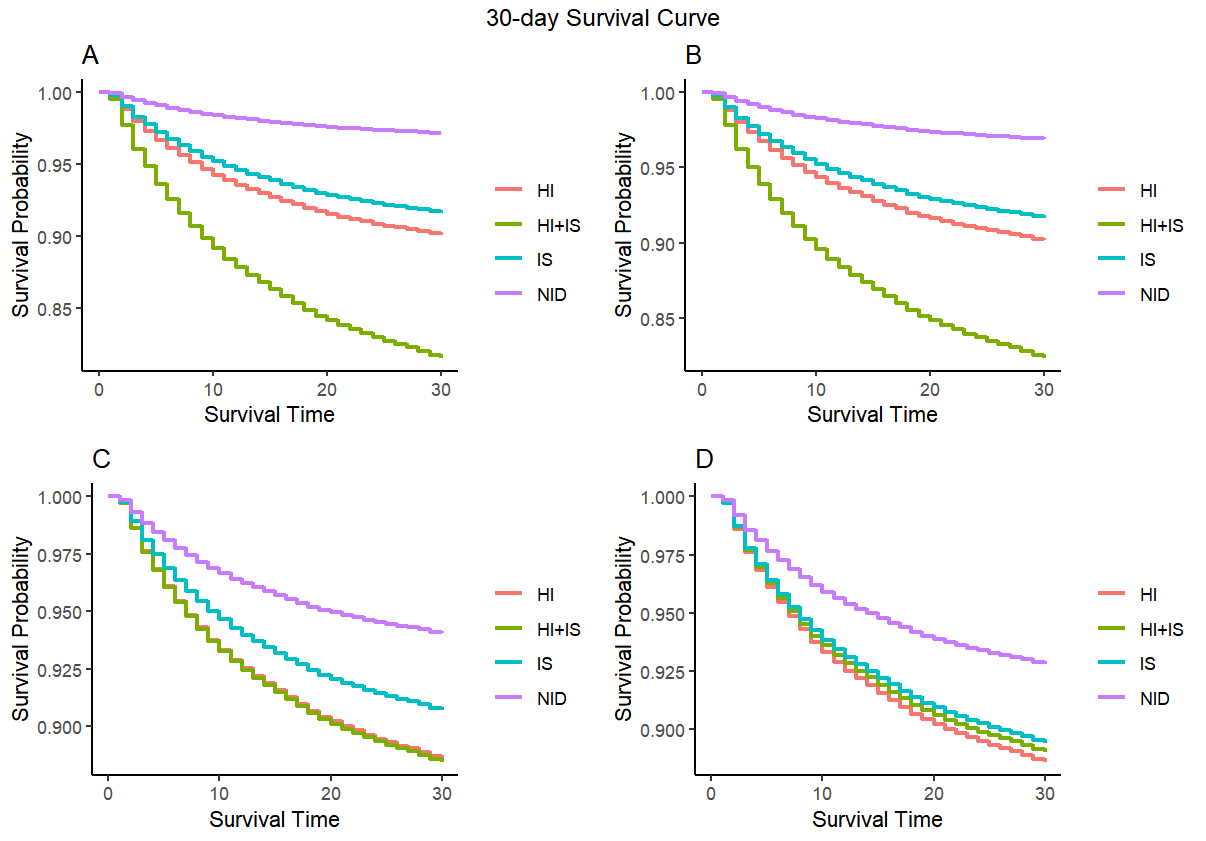


Supplementary Figure 4: Kaplan-Meier survival curves of 30-day mortality between subgroup.A: model 1 (uncorrected variable); B: model 2 (adjusted for age, gender, race); C: model 3 (adjusted for age, gender, race, APACHE II, charlson, sepsis, spetic shock, acute pancreatitis, myocardial infarct, HIV infection, ARDS, AKF, diabets, hypertenion, cerebrovascular disease, congestive failure, malignant cancer; D: model 4 (adjusted for all variable).


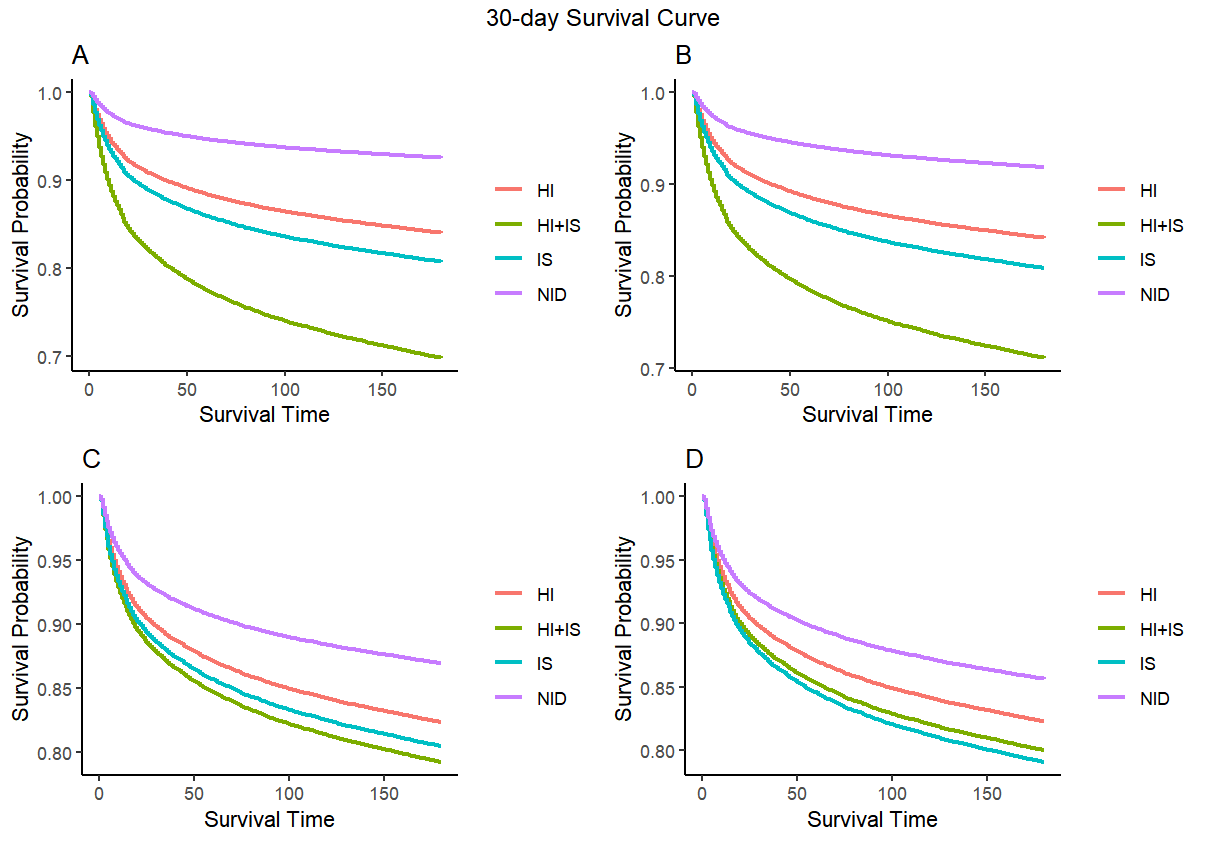


Supplementary Figure 5: Kaplan-Meier survival curves of 180-day mortality between subgroup. A: model 1 (uncorrected variable); B: model 2 (adjusted for age, gender, race); C: model 3 (adjusted for age, gender, race, APACHE II, charlson, sepsis, spetic shock, acute pancreatitis, myocardial infarct, HIV infection, ARDS, AKF, diabets, hypertenion, cerebrovascular disease, congestive failure, malignant cancer; D: model 4 (adjusted for all variable).
